# In Silico and In Vitro Study of the Bromelain-Phytochemical Complex Inhibition of Phospholipase A2 (Pla2)

**DOI:** 10.3390/molecules23010073

**Published:** 2018-01-19

**Authors:** Fatahiya Mohamed Tap, Fadzilah Adibah Abd Majid, Hassan Fahmi Ismail, Tet Soon Wong, Kamyar Shameli, Mikio Miyake, Nurul Bahiyah Ahmad Khairudin

**Affiliations:** 1 Malaysia Japan International Institute of Technology, Universiti Teknologi Malaysia, Jalan Sultan Yahya Petra, Kuala Lumpur 54100, Malaysia; fatahiyamohdtap@yahoo.com (F.M.T.); kamyar@utm.my (K.S.); miyakejaist@gmail.com (M.M.); 2 Institute of Marine Biotechnology, Universiti Malaysia Terengganu, Kuala Terengganu 21030, Malaysia; 3 Department of Bioprocess and Polymer Engineering, Faculty of Chemical and Energy Engineering, Universiti Teknologi Malaysia, Johor Bahru 81310, Malaysia; hassanfahmiismail@yahoo.com (H.F.I.); daniel_wongts@yahoo.com (T.S.W.)

**Keywords:** combination index, bromelain, phospholipase A2, anti-inflammatory agent, phytochemical

## Abstract

Phospholipase A2 (Pla2) is an enzyme that induces inflammation, making Pla2 activity an effective approach to reduce inflammation. Therefore, investigating natural compounds for this Pla2 inhibitory activity has important therapeutic potential. The objective of this study was to investigate the potential in bromelain-phytochemical complex inhibitors via a combination of in silico and in vitro methods. Bromelain-amenthoflavone displays antagonistic effects on Pla2. Bromelian-asiaticoside and bromelain-diosgenin displayed synergistic effects at high concentrations of the combined compounds, with inhibition percentages of more than 70% and 90%, respectively, and antagonistic effects at low concentrations. The synergistic effect of the bromelain-asiaticoside and bromelain-diosgenin combinations represents a new application in treating inflammation. These findings not only provide significant quantitative data, but also provide an insight on valuable implications for the combined use of bromelain with asiaticoside and diosgenin in treating inflammation, and may help researchers develop more natural bioactive compounds in daily foods as anti-inflammatory agent.

## 1. Introduction

Inflammation is a complex process involving many cell types and it acts as a body-protective response to injury, infections, cell damage and irritants. An inflammatory disorder occurs when inflammation becomes uncontrolled and causes damage to healthy tissues. For example, the inflammation pathway in adipose tissues is used for energy storage and lipid homeostasis, but the imbalance of food intake and expenditure leads to obesity. The discovery of obesity as an inflammatory state prompted researchers to explore the mechanism of inflammation related to obesity. Thus, researchers have investigated anti-inflammatory agents for the treatment of obesity. Bromelain was proven to be anti-inflammatory inhibitor by irreversibly inhibiting 3T3-L1 in adipogenesis by reducing adipogenic gene expression and inducing apoptosis and lipolysis in mature adipocytes [1].

Phospholipase A2 (Pla2) is a rigid tertiary structure enzyme with approximately eight disulfide bonds that can protect from heat denaturation and proteolysis [2]. This enzyme hydrolyzes the sn-2 ester bond of glycerophospholipids for the liberation of free fatty acids and lysophospholipids molecules [3]. To date, eleven Pla2 compounds belonging to groups IB, IIA, IIC, IID, IIE, IIF, III, V, X, XIIA, and XIIB have been characterized in mammals. The IIA group has been identified to contribute to the initiation of inflammation which subsequently induces the section of arachidonic acid and leads to the formation of inflammation mediators [4]. Arachidonic acid is metabolized either by the cyclo-oxygenase or lipo-oxygenase enzymatic pathway for the production of diverse families of eicosanoids such as prostaglandins and leukotrienes, which are primarily involved in the inflammatory pathway [4]. Meanwhile in reptiles, venomous snakes secret three groups of Pla2 enzymes, including IA, IIA, and IIB, which are responsible for several pathological manifestations of envenomation, such as inflammation and toxicity [4]. An excess level of Pla2 promotes multiple diseases related to vascular inflammation, such as coronary artery disease and acute coronary syndrome [5]. 

Recent studies have discovered several natural compounds that possess inhibitory activity against Pla2 [6,7]. However, treatment with a single anti-inflammatory drug often causes multiple adverse effects such as hepatoxicity, gastrointestinal haemorrhage, meningitis and asthma [8]. Therefore, a novel strategy combining two or more potential compounds that exhibit inhibitory effects against Pla2 activity has been proposed to overcome the problem. Additionally, this approach has previously been shown to enhance the thermal stability of the combination [9]. 

Bromelain and phytochemicals like amenthoflavone, asiaticoside, and diosgenin have been reported to exhibit inhibitory effects against Pla2 activity [7,10,11,12]. Bromelain, asisticoside and diosgenin appear to be safe compounds, as they do not show any toxic effects with a lethal dose (LD_50_) of up to 750 mg/kg in dogs, 50 mg/kg in mice and more than 8000 mg/kg in mice, respectively [13,14,15]. It was also discovered that the combination of phytochemical compounds with bromelain could enhance the functional properties and thermal stability and increase the shelf life of pineapple juice [9,16]. The combinations with natural products, including bromelain, was also proven to enhance the effect of other anti-inflammatory drugs such as paracetamol in the relief of the knee joint pain [17]. The effect of the combination between bromelain and antibiotics was shown to be more effective compared to antibiotics alone in the treatment of pneumonia, bronchitis and cellulitis [18].

In this study, the synergistic potential of combinations of bromelain and phytochemicals namely, amenthoflavone, asiaticoside, and diosgenin, against Pla2 was quantified. The combinations of bromelain-amenthoflavone (Br-Am), bromelain-asiaticoside (Br-As), and bromelain-diosgenin (Br-Di) were analyzed using a protocol developed by Chou and Talalay [19], which is widely used to determine the synergistic and antagonistic effects in combination studies. Subsequently, proof of the utility of the bromelian-phytochemical complex were generated by measuring the inhibitory activity against Pla2.

## 2. Results

### 2.1. Top Ten Ranking Compounds and Molecular Docking Results

**Figure 1:**
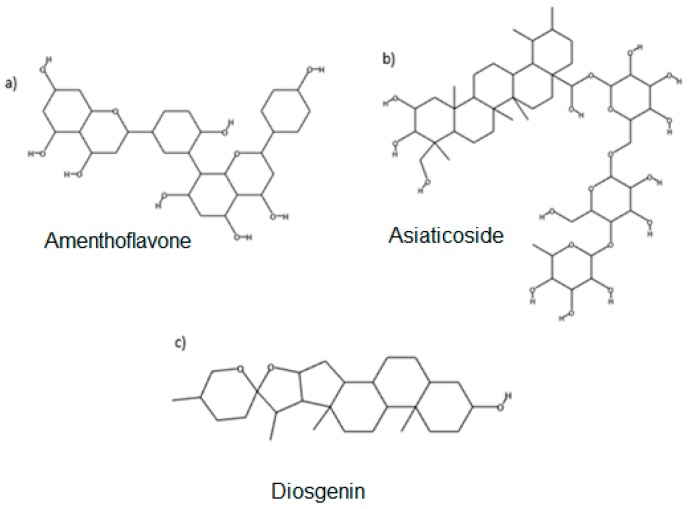
Chemical structures of the selected compounds: (**a**) amethoflavone; (**b**) asiaticoside; (**c**) diosgenin.

The current study aimed to select and investigate the natural compounds isolated from Malaysian herbs (Mherb) that are able to form strong and stable bonds with bromelain using a virtual screening approach. Final docked conformations had ranked 10 NADI compounds from highest to lowest ∆G of the binding energy as listed in Table 1. The more negative the ∆G values, the stronger the binding between a protein and a ligand. Among these 10 compounds only three compounds—amenthoflavone, asiaticoside and diosgenin—were selected for further preliminary experimental validation. Figure 1 shows the chemical structure of these compounds. The selection of these three compounds was based on the availability of the compounds.

**Figure 2:**
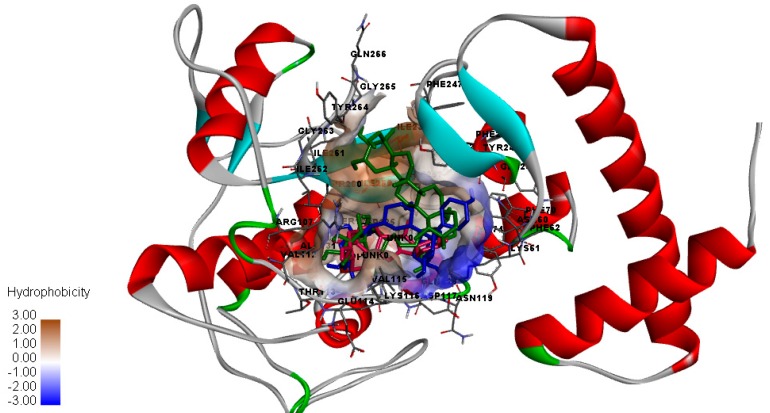
Surface representations of the binding site of bromelain model with the bound ligands, green: diosgenin, blue; amenthoflavone, and pink; asiaticoside shown in stick model.

Figure 2 shows the binding site for the docked conformation of bromelain with amenthoflavone, asiaticoside and diosgenin. It was seen that these compounds bound at the same binding region, indicate a similar potential for stabilization of the enzyme. Even though these compounds bound at the same binding region, they showed somewhat different binding orientations. The hydroxyl (OH) substituent was found to interact with polar residues, while the aromatic rings of the compounds were found to be buried in the hydrophobic site.

**Figure 3:**
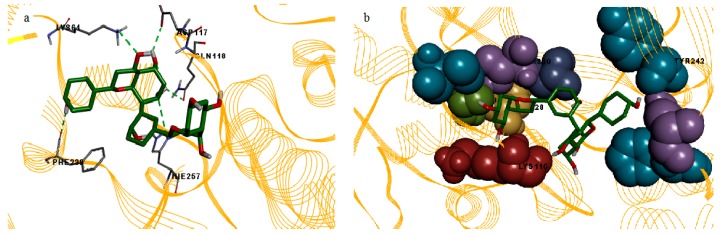
Illustrations of the hydrogen bonds and hydrophobic contacts between bromelain and amenthoflavone. (**a**) Hydrogen bond interactions between amenthoflavone (stick) and bromelain model (line ribbon). Residues that involved in hydrogen bond interactions are shown in stick model; (**b**) hydrophobic interactions between amenthoflavone (stick) and bromelain model (line ribbon). Residues that involved in hydrophobic interactions are shown in CPK model.

**Figure 4:**
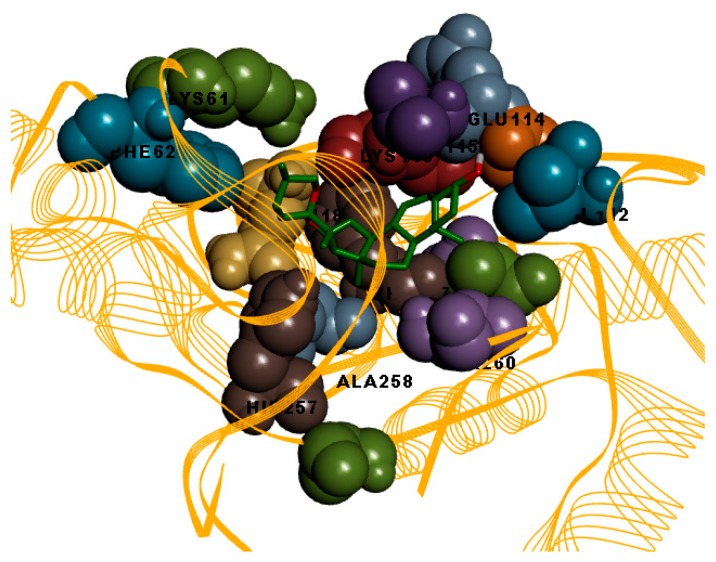
Illustration of the hydrophobic interaction between diosgenin (stick) and bromelain model (line ribbon). Residues that involved in hydrophobic interaction are shown in CPK model.

In this study, the binding mode patterns of the compounds were investigated based on their hydrogen bonding and hydrophobic interactions. Amenthoflavone formed eight hydrogen bonds and nine hydrophobic interactions (Figure 3). However there was no occurrence of hydrogen bond for the bromelain and diosgenin complex, but diosgenin formed hydrophobic interactions with fourteen bromelain residues which were Lys61, Phe62, Val112, Thr113, Glu114, Val115, Lys116, Gln118, Cys124, Phe127, Ala131, His127, Ala258, and Thr260 (Figure 4).

**Figure 5:**
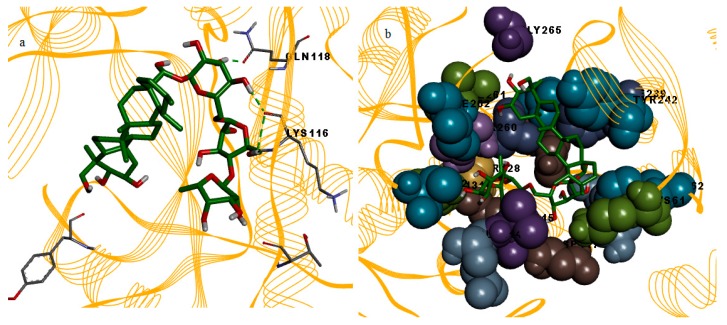
Illustrations of the hydrogen bonds and hydrophobic contacts between bromelain and asiaticoside. (**a**) Hydrogen bond interactions between asiaticoside (stick) and bromelain model (line ribbon). Residues that involved in hydrogen bond interactions are shown in stick model; (**b**) hydrophobic interactions between asiaticoside (stick) and bromelain model (line ribbon). Residues that involved in hydrophobic interactions are shown in CPK model.

All these residues were almost the same as the residues that formed hydrophobic contacts with amenthoflavone. Diosgenin did not mediate any hydrogen bonds, and the binding energy between this compound and bromelain was found to be the lowest. This is probably due to the formation of the hydrophobic contacts which contributed to the stability of the docked conformation between bromelain and diosgenin. Figure 5a showed the hydrogen bond contacts of asiaticoside with residues Thr113, Lys116, Gln118 and Tyr264 of bromelain. Asiaticoside also formed a network of hydrophobic contacts with residues Lys61, Phe62, Tyr74, Val112, Glu114, Val115, Asp117, Phe127, Ser128, Ala131, Ile232, Phe239, Tyr242, His257, Ile259, Ile261, Gly265, THR260, and Ile262 (Figure 5b). Asiaticoside showed the highest numbers of hydrophobic contacts. It is also the bulkiest structure compared to the other two compounds.

### 2.2. Inhibitory Activity of Bromelain, Amenthoflavone, Asiaticoside, and Diosgenin Against Pla2

**Figure 6:**
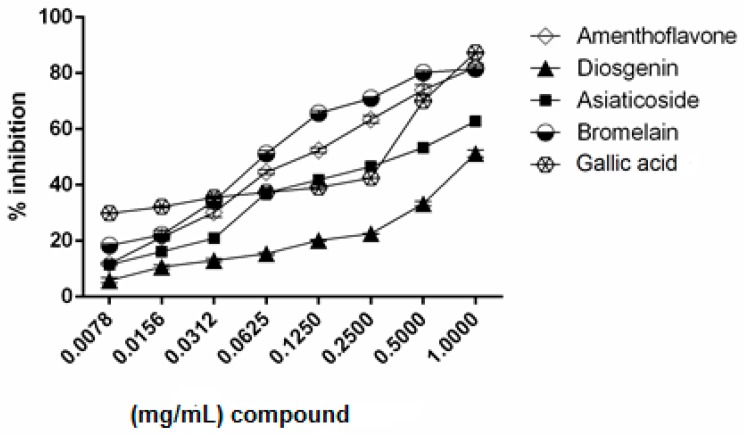
Inhibition of Pla2 activity by bromelain, amenthoflavone, asiaticoside, and diosgenin.

The selected compounds were further evaluated for their inhibitory activity of Pla2. As presented in Figure 6, all tested compounds showed a dose-dependent inhibitory activity against Pla2. Gallic acid was used as a positive control in order to support the method used for Pla2 inhibition. Bromelain and amenthoflavone at 1 mg/mL produced the highest inhibitory of 80% and 83%, respectively, followed by asiaticoside (60%) and diosgenin (45%).

The dose effect of bromelain, amenthoflavone, asiaticoside and diosgenin was applied in a median-effect plot for the determination of the following potential parameters: median effect dose at 50% inhibition (*D_m_*), slope of median effect plot (*m*), and linear correlation coefficient (*r^c^*) in Pla2 inhibitory activity. Table 2 shows that the *D_m_* values of bromelain, amenthoflavone, asiaticoside, and diosgenin were 0.0723, 0.1078, 0.3143 and 0.9659 mg/mL, respectively. The *r* values were greater than 0.95, indicating that this in vitro study is acceptable. The *D_m_* and *m* values for a single compound and their combination mixture were used for the calculation of CI through Equation (5) in Section 4.5 below.

### 2.3. Combined Effects of Phytochemicals and Bromelain Against Pla2

**Figure 7:**
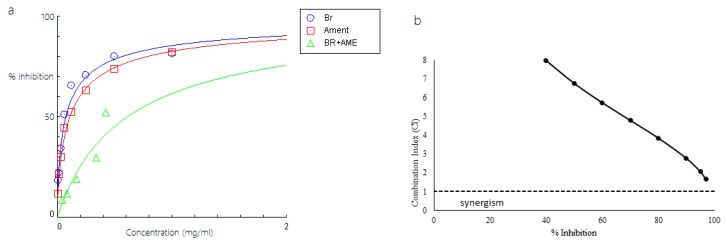
(**a**) Dose-response curve of bromelain (Br), amenthoflavone (Ament) and bromelain-amenthoflavone complex (BR + AME); (**b**) CI of bromelain and amenthoflavone on inhibition of Pla2.

**Figure 8:**
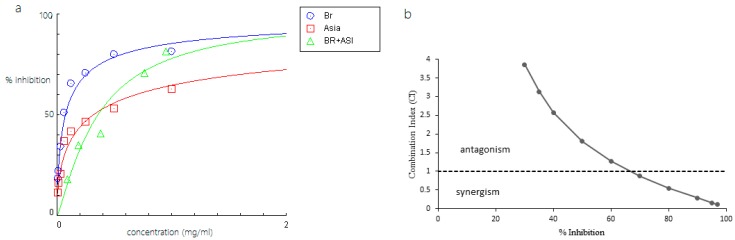
(**a**) Dose-response curve of bromelain (Br), asiaticoside (Asia) and bromelain-asiaticoside complex (BR + ASI); (**b**) CI of bromelain and asiaticoside on inhibition of Pla2.

**Figure 9:**
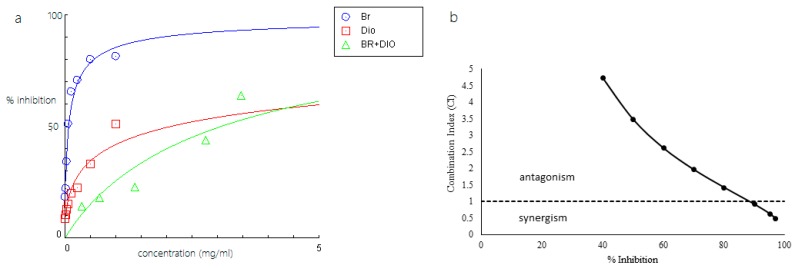
(**a**) Dose-response curve of bromelain (Br), disogenin (Dio) and bromelain-diosgenin complex (BR + DIO); (**b**) CI of bromelain and disogenin on inhibition of Pla2.

Among the combinations, Br-As showed the highest inhibitory activity because it required the lowest concentration (0.311 mg/mL; Table 2). The concentration of the combination between bromelain and asiaticoside was less than that of the concentration at IC_50_ of a single compound (either bromelain or asiaticoside). We used dose-effect curves and CI methods to investigate the combined effects of Br-Am, Br-As, and Br-Di against Pla2, and the results are summarized in Figure 7, Figure 8 and Figure 9. Figure 7 shows that the combination of bromelain and amenthoflavone displayed an antagonistic effect on Pla2 at the concentration used in the experiment. The CI value was near the additive effect line when the percent inhibition and concentration of the complex was high. Figure 8 shows that the Br-As complex exhibited a synergistic effect when the inhibition percentage was more than 70%.

At the inhibition percentage of approximately 70%, the combination Br-As was additive. However, the combined effect changed to antagonism at low inhibition levels (<70%). Meanwhile, the Br-Di complex showed a synergistic effect when the inhibition percentage was more than 90%. According to the results in Figure 7, Figure 8 and Figure 9, the combinations of bromelain with amenthoflavone, asiaticoside, or diosgenin were synergistic at high concentrations and antagonistic at low concentrations.

DRI values (Table 3) indicated that the combination of Br-As and Br-Di showed a favorable combination from IC_50_ to IC_95_ and IC_90_ to IC_95_, respectively. Meanwhile, no favorable DRI value was obtained from the combination between bromelain and amenthoflavone.

## 3. Discussion

Molecular docking is a powerful bioinformatics tool to gain insight into the most probable binding conformation of phytochemicals, which in return helps explain the reasons for their potency. Observing the structural features such as the position of aromatic rings and hydroxyl substituents (OH) on phytochemicals is essential in order to further understand the molecular interactions. Furthermore, the orientations of each compounds in the binding site are influenced by the hydrophobic and hydrogen bond interactions. Compounds that have more aromatic rings tend to interact with hydrophobic residues, while, compounds that have more OH substituents tend to form more hydrogen bonds. For example, amenthoflavone and asiaticoside formed more hydrogen bonds due to their bulkier structures that contain more OH substituents. The docking results also indicated that the active compounds can be stabilized in the binding site through hydrophobic interactions. Bdira and co-workers reported similar results suggesting the hydrophobic interactions with an aliphatic tail can influence protein stabilization and rigidification [20].

Bromelain, an enzyme that found abundantly in pineapple has been suggested as one of the most effective anti-inflammation agents with adequate toxicology and safety testing data as well as multiple biological activities [13]. However, bromelain is degraded at high temperatures, thus affecting the enzyme efficiency. The hydrophobic interactions produced a rigid protein structure which is difficult to denature by high temperature. Our results displayed the binding pocket of bromelain was surrounded by mostly hydrophobic residues and for that reason, all three of the compounds formed hydrophobic contacts in the binding pocket. This rationalized the study hypothesis regarding the interactions of phytochemicals and bromelain could enhance the stability of bromelain and increase the shelf lifetime. Furthermore, all tested compounds were favored to form hydrophobic conditions due to the presence of aromatic rings. These compounds bound in the hydrophobic pocket of bromelain, thus forming rigid hydrophobic-hydrophobic interactions. Collectively, this study suggested that the high thermal stability of bromelain-phytochemical complex was a result of the presence of hydrophobic-hydrophobic interactions.

Compounds that enhance protein thermostability have been associated with anti-inflammatory activities [21]. Based on the docking results, the developed bromelain-phytochemical complexes were subsequently analysed for its anti-inflammatory activity against Pla2. The results can facilitate the discovery of drug combinations that have a synergistic effect against the Pla2 activity. Bromelain–diosgenin complex presented a promising synergistic effect, with 90% inhibition of Pla2 despite the low anti-inflammatory activity of diosgenin. It showed a substantial additive effect when combined with bromelain. Meanwhile, the bromelain-asiaticoside complex displayed the most synergistic combination as the synergism was initiated at 70% inhibition. The DRI value for this combination required 1.12-fold less bromelain and 1.07-fold less asiaticoside at 50% inhibition. This value could be used as a guideline for the reduction of the toxicity effect on normal tissues and maximize the therapeutic effect. Unfortunately, antagonistic effect was observed on the combination of bromelain and amenthoflavone, possibly due to the mechanism of these compounds toward Pla2. Synergistic effects of natural product combinations can be affected by several factors such as the pharmacokinetics, physicochemical properties, complex multitarget effects and therapeutic approaches [22].

Natural compounds with good inhibitory activity on Pla2 and low cytotoxicity are useful for potential therapeutic applications. The anti-inflammatory activity of plant extracts above 70% at 250 mg/mL concentration is considered significant [23]. Of the three combinations, only Br-As and Br-Di led to dose-dependent Pla2 inhibition. Moreover, the combinations between bromelain and polyphenolic compounds enhanced the thermal stability of bromelain [9]. Furthermore, bromelain alone was easily denatured when the temperature is more than 50 °C, but the compound containing bromelain and polyphenol remained stable at this temperature. Therefore, the combinations between bromelain and phytochemical are potential anti-inflammatory agents. The synergistic effect of many compounds in natural products also enhanced their antioxidant activities because antioxidant compound will scavenge the free radicals produced from inflammation [24] and anti-inflammatory agents modulate the activities of proinflammatory enzymes and cytokines. During inflammation, eicosanoids, as proinflammatory mediators, are synthesized through Pla2 hydrolysis. The inhibitory activity of eicosanoid production from natural compounds may be induced by the inhibition of Pla2 enzyme activity. Many natural compounds are known inhibitors of Pla2 expression [25,26]. Meanwhile, the inhibitory activity of the combination between bromelain and phytochemical compounds on Pla2 remains unreported. Thus, the combination among Br-Am, Br-As, and Br-Di was selected because of the role bromelain plays in inflammation treatment and enhancement of functional properties when combined with phytochemical compounds. This combination can display synergistic activity and increase the therapeutic efficacy. Alternatively, these combinations; Br-Am, Br-As, and Br-Di might be used as food sources for treating inflammation instead of typically used synthetic drug therapy. It would be interesting to further investigate the effects of these complexes as the anti-inflammatory agent on a simple cell system such as mouse macrophages for evaluation purposes.

The intake of pineapple juice rich in bromelain can be potentially used as a therapy for inflammation disorders. Meanwhile, the combination between bromelain and phytochemicals that showed synergistic effect might be useful in treating inflammation because of the stability of bromelain and phytochemical complexes. This stability is useful in preserving the lifetime of the mixture. Furthermore, the shelf life of pineapple juice (source of bromelain) and polyphenol mixture is longer compared with that of pineapple juice alone [16]. Polyphenols act as antioxidant agents stabilizing the pineapple juice. Bromelain alone tends to be easily oxidized and denatured without an antioxidant agent. Dietary interference wherein naturally bioactive compounds in daily foods are used might be a more practical approach than the application of chemical therapy. Intake of asiaticoside and diosgenin accompanied by a certain amount of bromelain might be more efficient compared with these compounds alone because some phytochemicals are poorly absorbed and distributed in therapeutic application. Thus, the poor bioavailability of phytochemicals affects the effective dose delivered to the target cells/protein [27]. Even though natural products are typically known to be safe, it is challenging to determine the efficacy profile of these products due to the increment of the toxicity effects [28]. One of the implications of this study is that various experiments must be conducted to determine the optimum concentration of the combination complexes that are safe for use as a treatment for a disease. This combination must be done up to in vivo study in order to validate the efficiency of the combinations.

## 4. Materials and Methods

### 4.1. Materials

Bromelain, diosgenin, asiaticoside, porcine group IB phospholipase A2 (pG-IB), egg yolk phosphatidylcholine, red phenol, sodium taurodeoxycholate (NaTDC), and potassium phosphate were purchased from Sigma-Aldrich (St. Louis, MO, USA). Amenthoflavone was purchased from Atkins Chemicals (Chengdu, China).

### 4.2. Virtual Screening

#### 4.2.1. Protein and Ligand Library Preparation

The homology model of bromelain molecule was developed in previous research. The bromelain model was prepared using Autodock Tool (ADT) [29]. Hydrogen atoms were added to the receptor using ADT. Kollman charges were assigned and solvation parameters were added to this enzyme molecule. The grid binding box was centered at 48 × 167 × 63 and the size created with the dimensions of 34 × 36 × 32 with the default spacing is 1 Å. AutoGrid was used to create the affinity grids which focus at the active sites. The three dimensional (3D) structures of 4071 Malaysian natural compounds were obtained from the NADI database (www.nadi-discovery.com). Structures of these compounds were generated using Schrödinger’s LigPrep module [30]. In brief, protonation states were assigned at pH 7.0 using Epik. For each compound, LigPrep identified all tautomeric, one stereoisomeric state, and one low energy ring conformation. The structure were optimized using OPLS_2005 force field. NADI database were prepared for docking calculations using Raccoon [31] which added the Gasteiger-Marsili chargers, merged the non-polar hydrogen atoms onto their respective heavy atoms and generate the “pdbqt” docking input format for each compound.

#### 4.2.2. Molecular Docking

The virtual screening and docking was performed using AutoDock Vina [32]. Autodock Vina was used due to its accuracy and speed. AutoDock Vina was utilized to automate the docking process towards the NADI compounds. The predicted binding energy (∆G), which indicates the strength of compounds bind to the receptor is calculated based on scoring function used in Autodock Vina. The top ten docking conformations for each compound was selected using a Python script file. The selection was based on lowest energy binding. The H-bond, and hydrophobic interaction were analysed using ligplot server [33] and viewed using and Discovery studio visualizer.

### 4.3. Inhibition of Pla2 Activity

The inhibitory activity of Pla2 was tested according to the method described by De Aranjo and Radvany [34]. Briefly, the substrate consisted of 3.5 mM lecithin, a mixture of 3 mM NaTDC, 100 mM NaCl, 10 mM CaCl_2_, and 0.055 mM red phenol as colorimetric indicator, and 100 mL H_2_O. The pH of the reaction mixture was adjusted to 7.6. 0.2 μg of pG-IB was solubilized in 10% acetonitrile at a 0.002 μg/μL concentration. A volume of 2 μL of Pla2 solution was incubated with 2 μL of sample for 20 min at room temperature. Then, 200 μL of Pla2 substrate was added to the solution. Kinetic hydrolysis was performed for 5 min, and optical density was read at 558 nm. The Pla2 inhibitory activity was expressed in inhibition percentage and was calculated as follows:

(1) $$Enzyme\;activity=\frac{OD_{zero}-\;OD_{15\;\min}}{15\;\min}$$

(2) $$\%\;inhibition=\frac{enzyme\;activity_{-ve\;control}-\;enzyme\;activity_{sample}}{enzyme\;activity_{-ve\;control}}\;\times \;100$$

### 4.4. Experimental Design of Combination Study

An experimental design was formulated for the evaluation of the effect of phytochemical compounds (amenthoflavone, asiaticoside, and diosgenin) combined with bromelain. First, the IC_50_ values of amenthoflavone and bromelain were calculated on the basis of dose-response curves. Subsequently, six concentrations of amenthoflavone at 2.5 IC_50_, 2 IC_50_, IC_50_, 0.5 IC_50_, and 0.2 IC_50_ was prepared and combined with equal concentration (2.5 IC_50_, 2 IC_50_, IC_50_, 0.5 IC_50_, and 0.2 IC_50_) of bromelain at equal volume. The inhibitory effects of combined bromelain and amenthoflavone were examined on Pla2. The same experimental design described above was repeated for combination of Br-As and Br-Di. Table 4 shows the series of concentration of each bromelain-phytochemical complex.

### 4.5. Median Effect, Combination Index (CI), and Dose Reduction Index Analysis

To correctly estimate the combination effects (synergism, additive, and antagonism) of compounds, we applied the median-effect principle and CI developed by Chou and Talalay [19]. The median-effect equation is as follows:

(3) $$\frac{f_{a}\;}{\;f_{u}}={\left(\frac{D}{D_{m}}\right)}^{m}$$

In Equation (1), *D* is the dose, *D_m_* is the dose required for 50% inhibition, *f_a_* is the fraction effected by dose *D*, *m* is a coefficient of the sigmoidicity of dose-effect curve, and *f_u_* is the unaffected fraction (*f_a_* = 1 − *f_u_*). A rearrangement of Equation (1) gives:

(4) D=Dm[fa/(1− fa)]1m

*CI* is expressed as:

(5) $$CI=\frac{{\left(D\right)}_{1}}{{\left(D_{x}\right)}_{1}}+\;\frac{{\left(D\right)}_{2}}{{\left(D_{x}\right)}_{2}}$$

We used the dose reduction index (DRI) to determine whether the combination can result in a reduction-compound dose. Dose reduction is important because it can reduce toxicity and maintain or increase the main therapeutic efficacy. Favorable DRI values would be >1, whereas unfavorable ones would be <1.

### 4.6. Statistical Analysis

All data were expressed in mean ± SEM. Statistical analysis was performed using the SPSS program (V.18.0, IBM Corp., Armonk, NY, USA) with one way ANOVA and Tukey test. Significant difference was considered as *p* < 0.05.

## Figures and Tables

**Table 1:** Top 10 compounds from the NADI database after virtual screening against bromelain.

Rank	Compound Name	Binding Affinity (∆G) (kcal/mol)	Plant
1	Α-Viniferin	−11.3	*Shorea gibbosa*
2	Gnetin E	−11.3	*Gnetum gnemonoside*
3	Isorhoifolin	−11.1	*Theobroma cacao*
4	Lupenone	−11.0	*Murraya paniculata*
5	Amenthoflavone	−11.0	*Garcinia prainiana*
6	Diosgenin	−10.8	*Costus speciousus*
7	Echinocyctic acid	−10.7	*Eclipta prostata*
8	Smilagenin	−10.7	*Trigonella foenum*
9	Asiaticoside	−10.7	*Centella asiatica*
10	Friedelin	−10.6	*Lawsonia inermis*

**Table 2:** Dose-effect relationship parameters of Br-Am, Br-As, and Br-Di combinations and single compounds against Pla2.

Compound	*D_m_* (mg/mL) ^a^	*m* ^b^	*r* ^c^
Bromelain	0.0723	0.6767 ± 0.0450	0.9879
Amenthoflavone	0.1079	0.4480 ± 0.0489	0.9968
Asiaticoside	0.3143	0.5258 ± 0.0381	0.9846
Diosgenin	0.9659	0.7018 ± 0.0229	0.9659
Bromelain (Br) + amenthoflavone (Am)	0.6093 (Br = 0.2437 mg/mL) (Am = 0.3655mg/mL)	-	-
Bromelain (Br) + asiaticoside (As)	0.311 (Br = 0.020 mg/mL) (As = 0.2911 mg/mL)	-	-
Bromelain (Br) + diosgenin (Di)	3.0603 (Br = 0.1500 mg/mL) (Di = 2.9103 mg/mL)	-	-

^a^*D_m_* is the median-effect dose required for 50% inhibition calculated from compusyn software; ^b^ *m* is the coefficient of the sigmoidicity/shape of dose-effect curve; ^c^ *r* is the significant coefficient that should be more than 0.95.

**Table 3:** DRI values of singles compound, Br-Am, Br-Aa, and Br-Di at 50%, 75%, 90% and 95% inhibition of Pla2.

Compound Combination	DRI Value ^+^			
	IC_50_	IC_75_	IC_90_	IC_95_
Bromelain (Br) + amenthoflavone (Am)	0.29 (Br) 0.29 (Am)	0.48 (Br) 0.44 (Am)	0.76 (Br) 0.68 (Am)	1.06 (Br) 0.90 (Am)
Bromelain (Br) + asiaticoside (As)	1.14 (Br) 1.07 (As)	2.36 (Br) 3.55 (As)	4.87 (Br) 11.7 (As)	8.00 (Br) 26.35 (As)
Bromelain (Br) + diosgenin (Di)	0.84 (Br) 0.70 (Di)	0.77 (Br) 2.58 (Di)	1.22 (Br) 9.42 (Di)	1.68 (Br) 22.73 (Di)

^+^ DRI represents the order of magnitude (fold) of dose reduction that is allowed in reduction. DR < 1 and DRI > 1 indicate non-favorable and favorable, respectively.

**Table 4:** The concentration of each bromelain-phytochemical complex.

Compounds	Concentration (mg/mL)				
	2.5 IC_50_	2 IC_50_	1 IC_50_	0.5 IC_50_	0.2 IC_50_
Bromelain	0.1808	0.1446	0.0723	0.03615	0.01445
Amenthoflavone	0.2698	0.2158	0.1079	0.0540	0.0216
Asiaticoside	0.7858	0.6286	0.3143	0.1572	0.0628
Diosgenin	2.4148	1.9308	0.9659	0.4829	0.1932
